# New Antiadhesive Hydrophobic Polysiloxanes

**DOI:** 10.3390/molecules26040814

**Published:** 2021-02-04

**Authors:** Maria Nowacka, Anna Rygała, Dorota Kręgiel, Anna Kowalewska

**Affiliations:** 1 Centre of Molecular and Macromolecular Studies, Polish Academy of Sciences, Sienkiewicza 112, 90-363 Łódź, Poland; mnowacka@cbmm.lodz.pl; 2 Department of Environmental Biotechnology, Faculty of Biotechnology and Food Sciences, Lodz University of Technology, Wólczańska 171/173, 90-924 Łódź, Poland; anna.rygala@p.lodz.pl (A.R.); dorota.kregiel@p.lodz.pl (D.K.)

**Keywords:** functionalized polysiloxanes, hydrophobic, antimicrobial properties

## Abstract

Intrinsic hydrophobicity is the reason for efficient bacterial settlement and biofilm growth on silicone materials. Those unwelcomed phenomena may play an important role in pathogen transmission. We have proposed an approach towards the development of new anti-biofilm strategies that resulted in novel antimicrobial hydrophobic silicones. Those functionalized polysiloxanes grafted with side 2-(carboxymethylthioethyl)-, 2-(*n*-propylamidomethylthioethyl)- and 2-(mercaptoethylamidomethylthioethyl)- groups showed a wide range of antimicrobial properties towards selected strains of bacteria (reference strains *Staphylococcus aureus*, *Escherichia coli* and water-borne isolates *Agrobacterium tumefaciens*, *Aeromonas hydrophila*), fungi (*Aureobasidium pullulans*) and algae (*Chlorella vulgaris*), which makes them valuable antibacterial and antibiofilm agents. Tested microorganisms showed various levels of biofilm formation, but particularly effective antibiofilm activity was demonstrated for bacterial isolate *A. hydrophila* with high adhesion abilities. In the case of modified surfaces, the relative coefficient of adhesion for this strain was 18 times lower in comparison to the control glass sample.

## 1. Introduction

Polysiloxanes (silicones) are an important class of inorganic polymers of high temperature and oxidative stability, excellent low temperature flexibility, high resistance to weathering and many chemicals, that has found many industrial and household uses [1,2,3]. Silicone materials exhibit also good biocompatibility, tolerance to sterilization, bio-durability and hemocompatibility [4,5]. That is the reason for their widespread application for medical purposes and in healthcare. Silicone-coated surfaces operate in both single-use and reusable applications, e.g., as artificial implants, catheters and drains or contact lenses. They are hypoallergenic and have a user-friendly appearance and feel. Due to the low rotation barriers around polar siloxane bonds, most poly(alkylsiloxanes) are very flexible [1,6]. Nonpolar side groups reduce the critical surface tension of polysiloxanes and make them capable of wetting most surfaces.

Despite those obvious advantages, polysiloxanes also have limitations and one of them is their intrinsic hydrophobicity [7] which, unfortunately, makes them attractive supports for bacterial settlement and biofilm growth [8,9,10]. Adhered bacterial cells in mature biofilms produce extracellular polymeric substances (EPSs) which promote the development of biofilm structures. Moreover, they usually express starvation phenotypes and defense mechanisms (antibiotic resistance or multidrug resistance). That is why traditional disinfectants may not be efficient enough to destroy microbial membranes. Moreover, positive selection of the most resistant cells may even enhance the biofilm adhesion.

Various polymeric materials (including silicones) may be modified to become biofilm-resistant and enhance their antibacterial properties [11,12,13,14]. Initial tests that were directed towards killing the microorganisms were mainly based on the use of toxic quaternary ammonium-based materials [15]. Moreover, sediments of dead cells that may become the source of secondary infections are an additional problem. Environmental concerns led to the development of new methods that provided long-term antimicrobial activity, hampering the growth of microorganisms while not adversely affecting the properties and stability of synthetic polymers. Strategies that would improve the resistance of silicones towards biofilm formation and make them bacteria-repellent while preserving their biocompatibility became the main concern in studies on antimicrobial silicones of the new generation. For example, antimicrobial and antifouling activity was noted for polysiloxanes grafted with uncharged hydrophilic poly(ethylene glycol) (PEG) [16,17] and polyacrylate [18] chains as well as hydrolysable block polysiloxane-polymethacrylate copolymers [19]. Such amphiphilic polymers were designed as non-antibiotic antimicrobials of minimal toxicity to mammalian cells, and their antiadhesive activity can be linked to their hydrophobic/hydrophilic balance. Hydrophilic units improve the biocompatibility of macromolecules and their binding to polyanionic cell membranes while hydrophobic segments insert into the lipid bilayer and cause its reorganization and damage. More recent solutions for controlling biofilms by synthetic biology methods are based on the protein engineering of biofilm-related enzymes and secretion of signaling molecules for cell–cell communication (quorum sensing) [20]. The idea of grafting polysiloxanes with chemical entities that would mime components of natural quorum-sensing molecules seems to be an attractive alternative. The library of antimicrobial and bacteriostatic polysiloxanes was thus expanded by us and others for systems containing enzymes [21] as well as residues of *N*-acetylcysteine [22,23] and guanidine [24]. Those polymeric materials were hydrophilic and some of them were even fairly soluble in water.

**Scheme 1:**
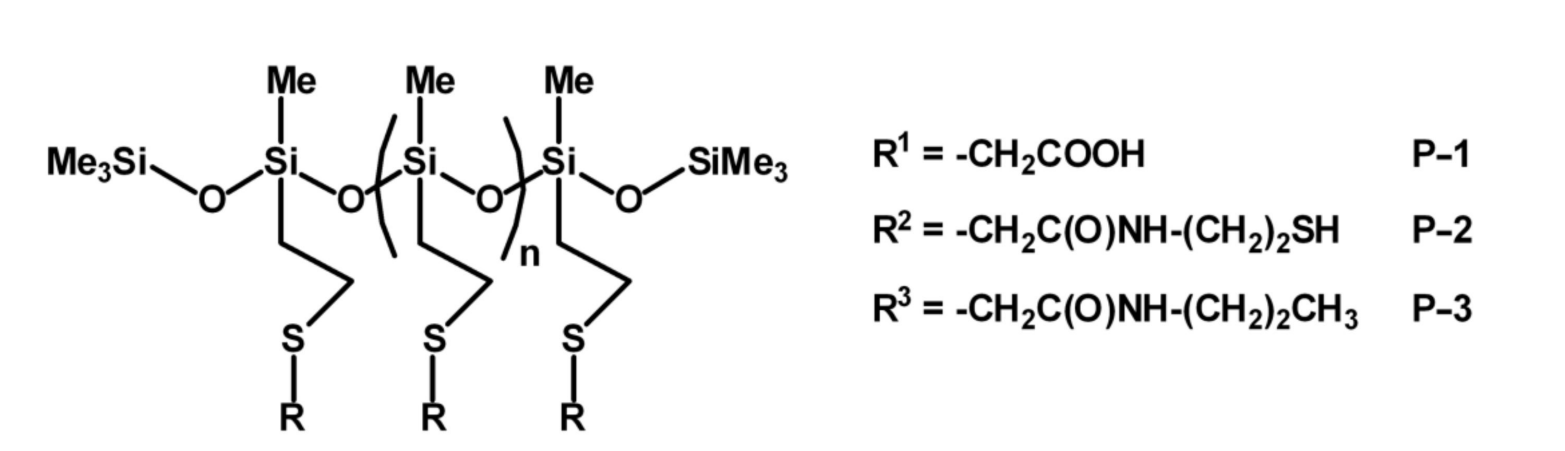
Structure of functionalized hydrophobic polysiloxanes of antimicrobial properties.

Looking for species that would exhibit targeted anti-biofilm activity, not changing the characteristic hydrophobicity of silicones, we have tested new polysiloxanes grafted with 2-(carboxymethylthioethyl)-(P-1), 2-(mercaptoethylamidomethylthioethyl)-(P-2) and 2-(*n*-propylamidomethylthioethyl)-(P-3) side groups (Scheme 1). The premise for this approach was the fact that carboxylic acids, including ibuprofen [25] and acetic acid [26,27], are regarded as non-antibiotic drugs and are well-known for their antimicrobial behaviour. Polysiloxanes with amide linkers in their side chains resemble, to some extent, antibacterial silicones modified with peptides [28,29]. The functionalization of polysiloxanes with mercaptoethyl residues was inspired by the well-known ability of thiols to take part in the disruption of proteins through oxidation into S–S bonds and/or SH-induced S–S interchange reactions [30,31]. Surface energy measurements proved that all the prepared polymers are hydrophobic. The materials were tested against a wide range of microorganisms (gram-positive and gram-negative bacteria, fungi and algae) that belong to different systematic groups (prokaryotes, eukaryotes) with different cell organizations and cell wall structures. Those microorganisms commonly occur in water environments. Most of them exhibit high adhesion abilities and are also responsible for nosocomial infections. Tests with selected bacterial strains (*Staphylococcus aureus, Escherichia coli, Agrobacterium tumefaciens, Aeromonas hydrophila*), fungi (*Aureobasidium pullulans*) and algae (*Chlorella vulgaris*) indicated good anti-microbial properties of the functionalized polysiloxanes. A considerable decrease in adhesion was observed in the amount of *A. hydrophila* cells, depending on the kind of functional groups that were grafted at polysiloxane chains. The obtained results show that certain active organic groups may be used for the valuable modification of hydrophobic silicones and improvement of their antimicrobial properties.

## 2. Results and Discussion

### 2.1. Synthesis and Properties of the Functionalized Polysiloxanes

Polymethylsiloxane homopolymers grafted with side 2-(carboxymethylthioethyl) groups (P-1) were obtained by the photoinitiated addition of thioglycolic acid to poly(vinylmethylsiloxanes), adapting the procedure described earlier for the preparation of poly(2-(carboxymethylthioethyl)silsesquioxanes) [32]. The quantitative reaction resulted exclusively in β-addition product, which was subsequently functionalized with 2-(mercaptoethylamido-methylthioethyl)-(P-2) and 2-(*n*-propylamidomethylthioethyl)methyl- (P-3) moieties through direct amidation of the carboxylic groups with, respectively, cysteamine and *n*-propylamine, using SiO_2_ as a heterogeneous, reusable catalyst. The environmentally benign silica catalyst effectively catalyzed amide synthesis from acids and amines without production of toxic by-products [33,34]. The degree of conversion was estimated following the relative increase in new signals corresponding to -CH_2_- in the products (3.5 ppm for P-2 and 1.7 ppm for P-3) with respect to the resonances of Si-Me groups in ^1^H-NMR spectra. The structure of products was characterized using ^13^C and ^29^Si-NMR and FTIR spectroscopies. Characteristic vibration modes proved the formation of the secondary amide bonds (amide I band at 1650 cm^−1^ (ν C = O) and amide II band at 1578 cm^−1^ (δ NH in plane + ν C-N)) as well as the presence of S-H groups (ν S-H at 2460 cm^−1^) in P-2. The conversion of COOH groups into amide bonds was not quantitative due to the steric congestion that becomes an obstacle once about 70 and 60% of substitution was reached in the case of P-2 and P-3, respectively. The reaction yield was 52–56%. The products were purified by three-fold precipitation in nonpolar solvents. Their properties were studied in comparison to those of the precursor VMS-T11.

**Figure 1:**
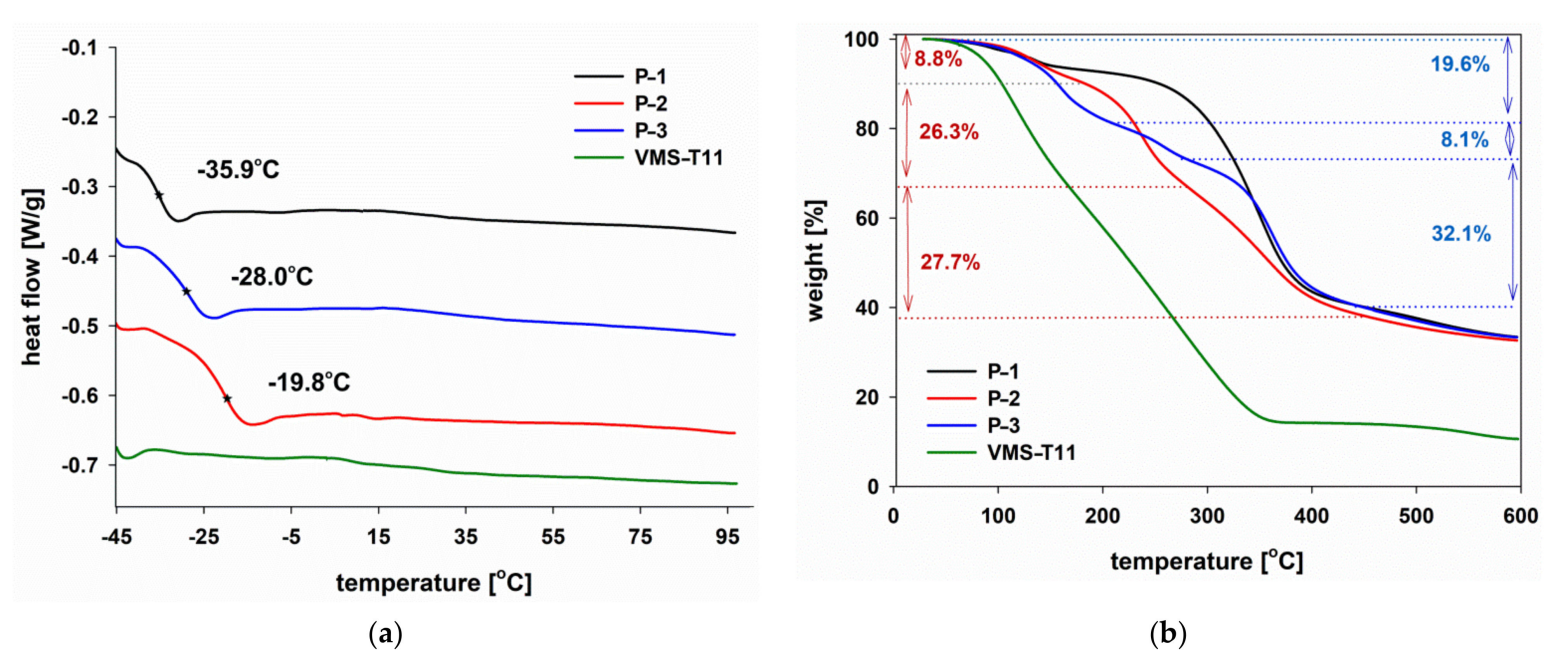
Thermal characteristics of the functionalized silicones: (**a**) Differential Scanning Calorimetry (DSC), 3rd heating run; (**b**) Thermogravimetric Analysis (TGA) in N_2_ 10 °C/min.

Calorimetric analysis of the obtained products showed (Figure 1a) that the characteristic temperature of devitrification recorded for all the studied polymers is well below 0 °C while being significantly higher than the characteristic glass transition temperature for poly(methylvinylsiloxanes) [35]. The effect may be correlated with the presence of hydrogen bonds between carboxylic groups or amide linkages in the side chains. The polymers are thermally stable up to 100 °C in the nitrogen atmosphere (Figure 1b, Table 1) which is significantly different to the rapid depolymerization of VMS-T11 (the first decomposition step with T_d1_ at 117 °C, at V_d1_ of 8.41%∙min/°C, followed by the slower degradation of the thermally crosslinked polysiloxane). Thermolysis of the siloxane precursor proceeds in a way typical of poly(alkylsiloxanes) (chain scission and backbiting) [36,37], although the ceramic residue (10 wt.%) suggests that the sample must have partly crosslinked at T > 350 °C through the radical polymerization of vinyl groups [38,39].

Thermal degradation of the studied products P-1, P-2 and P-3 starts at T > 120 °C. Within 120–150 °C, traces of moisture and volatiles may desorb from the material, as previously shown for ladder [2-(carboxymethylthio)ethyl]polysilsesquioxanes [40] and octahedral silsesquioxanes grafted with *N*-ethylhexanamide groups [41]. However, for P-3, the initial weight decrease overlaps with another process with a maximum at 159 °C (19.6 wt.% decrease at 1.7%∙min/°C). For P-1, the main degradation step (60% weight loss, T_d2_ = 350.3 °C, V_d2_ = 5.77%∙min/°C) occurred within 250–400 °C and may be correlated to the decarboxylation of side -COOH groups [42,43]. Thermal decomposition of P-2 and P-3 is more complicated due to the more complex composition of the side groups. For both P-2 and P-3, two additional phenomena may be distinguished: a weight loss within 200–300 °C and another one at 300–400 °C. Degradation of P-2 within 200–300 °C (T_d2_ = 238.2 °C, V_d2_ = 6.10%∙min/°C) may be linked to partial siloxane chain scission/chain transfer as a result of interactions of nucleophilic thiols and electrophilic silicon atoms in the main chain, analogously to the degradation pattern observed for silicones functionalized with γ-chloropropyl groups [44,45]. The next process observed for P-2 was also severe and quite rapid (T_d3_ = 357.4 °C, V_d3_ = 2.77%∙min/°C), and we have attributed it to amide bonds degradation (analogously to those reported in the literature [34,41,43,46]). The residual carboxylic moieties (~30% of the siloxane units) may also participate in the decomposition of amide bonds (acidolysis of amides [47,48]). The decomposition steps observed for P-3 during the thermal treatment overlap largely with those observed for P-2. However, the rate of decomposition at T_d3_ = 361.9 °C (V_d3_ = 5.80%∙min/°C) is larger than that observed for P-2, which may be attributed to the lack of reactive moieties that could induce crosslinking of the polysiloxane matrix. The ceramic residue at 600 °C was similar for all the studied functionalized silicones (33–34 wt.%).

**Figure 2:**
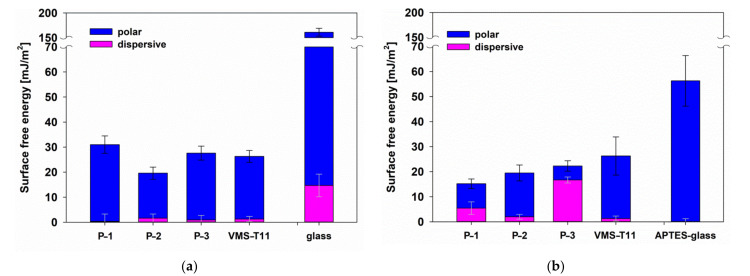
Surface free energy of functionalized polysiloxanes in comparison to their precursor VMS-T11 coated on (**a**) bare glass and (**b**) APTES-glass; (data presented as the mean ± standard deviation (SD, *n* = 4)).

Wettability of the studied polymers was estimated using the sessile drop technique for samples coated onto glass slides. Contact angles of the test liquids (water and glycerol) were measured at room temperature and free surface energy was determined (Figure 2). Despite the presence of hydrophilic units in the functional groups, the hydrophobicity of the studied samples was similar to that of poly(vinylmethylsiloxanes) [32,49]. Thin films of P-1, P-2 and P-3 reduced the surface free energy of both clean and 3-aminopropyltriethoxysilane (APTES)-modified glass supports with respect to the effect of VMS-T11 which indicates that, analogously to poly(vinyl-co-(2-hydroxyethylthioethyl))methylsiloxanes [49], the functional macromolecules adapted their conformation in order to form the most energetically favoured interface with hydrophobic air. The effect was found to be even more pronounced for P-1 coated on APTES-glass (the free surface energy of VMS-T11, P-2 and P-3 did not change upon changing the type of support). In this case, -COOH functional groups may be involved in the formation of hydrogen bonds and ionic structures with aminopropyl groups grafted on the surface of slides. As a result, the polymer chain adopts the most suitable conformation for those interactions, leaving Me groups more exposed to air, and thus causing the relative decrease in surface energy. The observed changes in free surface energy illustrate the ability of the functionalized polysiloxanes to adapt to the environmental conditions, including specific components of biofilms.

### 2.2. Antiadhesive Properties of the Functionalized Silicones

The functionalized polysiloxane materials were tested against a wide range of prokaryotic and eukaryotic microorganisms: selected bacterial strains (*S. aureus, E. coli, A. tumefaciens, A. hydrophila*), fungi (*A. pullulans*) and algae (*C. vulgaris*) with different cell organizations and cell wall structures. In our studies on cell adhesion, we decided to use a rather long, 6-day contact of the polymer with the tested microorganism. This strategy is not only most suitable for testing the anti-adhesive activity of a polymer surface, it also allows for evaluation of the reaction of microbial cells to the presence of functional groups grafted on the polysiloxane materials. A similar methodology was used in our previous research works on bioactive polymers [22,23]. The activity tests for the modified polysiloxanes were carried out in comparison to glass (control) and hydrophobic poly(vinylsilsesquioxane) (LPSQ) [50], which was used as a general model of hydrophobic silicone materials (instead of liquid VMS-T11) due to the higher durability of coatings formed on glass supports. The rapid and simple luminometric method that was chosen for this study allowed the estimation of biological material on the test surfaces. This method is based on bacterial ATP quantification and can be used not only to indirectly evaluate the total number of adhered cells, but also the cell viability, including cells that are unable to grow. This is worth noting, especially in the case of modified surfaces with potential antimicrobial activity.

**Figure 3:**
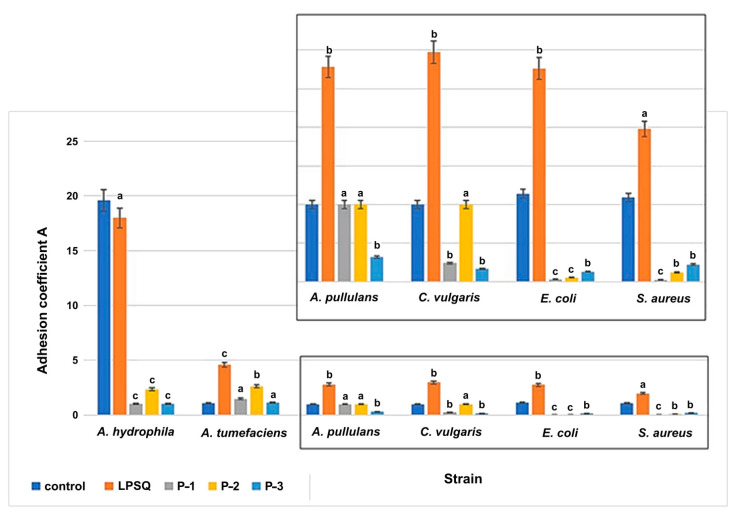
Adhesion coefficient (A) for microbial strains and polysiloxanes functionalized with reactive side groups and a model hydrophobic silicone (LPSQ). The results were compared to those for the control glass carriers. Values with letters show statistically significant differences: a, *p* ≥ 0.05; b, 0.005 < *p* < 0.05; c, *p* < 0.005.

Contrary to the hydrophobic LPSQ, all the functionalized polysiloxanes showed strong antimicrobial activities; however, their action depended on both the type of active group and the type of microorganism. Figure 3 presents the microbial abilities of cell adhesion after 6 days of incubation, expressed as the relative adhesion coefficient A. This parameter allows for estimation of the tendency of microbial cells to adhere, in relation to planktonic cells in the tested sample.

Significant differences were found by one-way ANOVA test in terms of adhesion results for the very adhesive bacterial strain of *A. hydrophila*. This water-borne isolate shows strong adhesive abilities, probably due to the presence of many peritrichous lateral flagella and non-pillar polysaccharide adhesins that play an important role in cell adhesion [51]. For this strain, the A coefficient in the presence of functionalized silicones was 18 times lower in comparison to the control glass sample. There is a striking difference in the adhesion of microorganisms on the surfaces of P-1, P-2 and P-3 in comparison to the model of a hydrophobic silicone analogue (LPSQ). All the tested functionalized polysiloxane materials also exhibited antimicrobial properties against gram-positive and gram-negative bacteria, fungi and algae that showed weaker adhesive abilities in comparison to *A. hydrophila* strain. The relative adhesion coefficient values for these microorganisms and glass carriers were lower and equal from 1 to 1.14. However, for strains with much weaker adhesion abilities, the differences between the results for the control glass sample and its modifications were not so significant. In general, the reference bacterial strains *E. coli* and *S. aureus* were found to be less adhesive than the wild-type strains *A. hydrophila* and *A. tumefaciens*. However, a significant difference can be observed comparing the behavior of *E. coli* and *S. aureus* on surfaces covered with P-1, P-2 and P-3 in comparison to the sample of hydrophobic LPSQ. P-1 seems to be the most effective, although the difference between P-1, P-2 and P-3 is not high. The qualitative effect of the modified polysiloxanes on *C. vulgaris* was similar to that on *A. tumefaciens*.

Our observations suggest that presence of bioactive groups: -CH_2_CH_2_SCH_2_COOH, -CH_2_CH_2_SCH_2_C(O)NHCH_2_CH_2_SH or -CH_2_CH_2_SCH_2_C(O)NHCH_2_CH_2_CH_3_ grafted to silicon atoms of polysiloxane chains may be responsible for the antimicrobial activity of these systems. The structural segments of those active groups are widely known for their antimicrobial activity. They may act alone or as a synergistic group against a wide range of microorganisms. [52]. Köllnberger and co-workers documented recently that carboxylic-acid-modified silicone materials exhibited a specific antimicrobial activity against clinically relevant pathogens [53]. The presence of thiol and/or methyl groups also markedly increases the potency of different antimicrobials [54,55].

**Figure 4:**
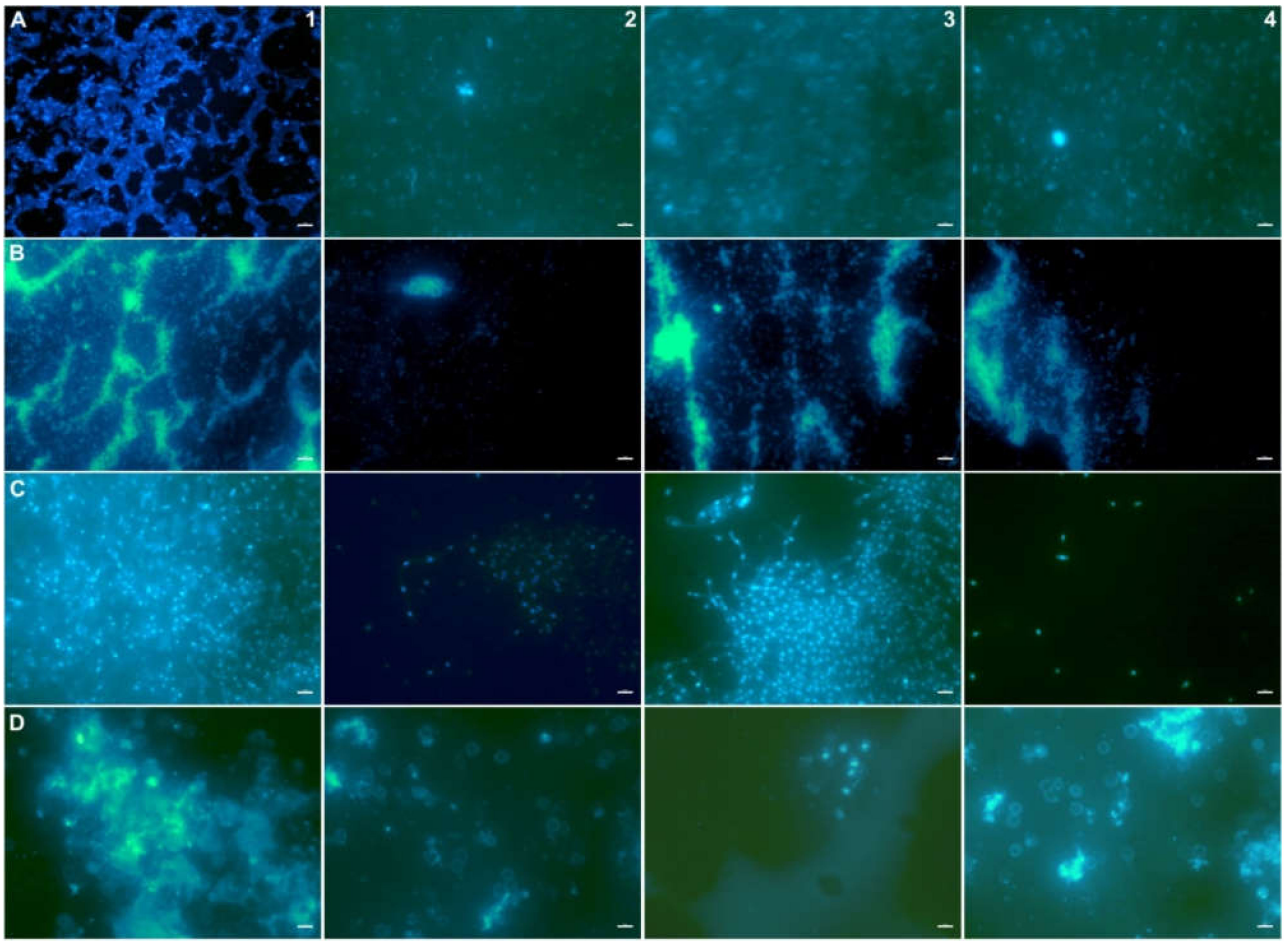
Microbial attachment observed by fluorescence microscopy: (**A**) *A. hydrophila* biofilms; (**B**) *A. tumefaciens* biofilms; (**C**) *A. pullulans* biofilms; (**D**) *C. vulgaris* biofilms; (**1**) control; (**2**) P-1; (**3**) P-2; (**4**) P-3 (scaling bar: 10 μm).

Microbial adhesion was also detected by microscopic observations (Figure 4). On modified surfaces, in the case of the *A. hydrophila* strain, groups of loosely packed cells were noticed. Therefore, this observation confirmed the results of luminometric studies. However, in the case of other tested microorganisms, the final effect varied and depended on the kind of microorganism.

*A. tumefaciens* rods are often isolated from water environments; they show the ability to create various exopolysaccharides that play a major role in biofilm formation. These compounds, namely cyclic-β-(1,2)-glucan, cellulose, curdlan, succinoglycan, and other unipolar polysaccharides, protect bacteria against environmental stresses [56]. The cell wall of microalgae and fungi also consists of a polysaccharide and glycoprotein matrix providing the cells with a formidable defense against different environmental stress conditions.

**Figure 5:**
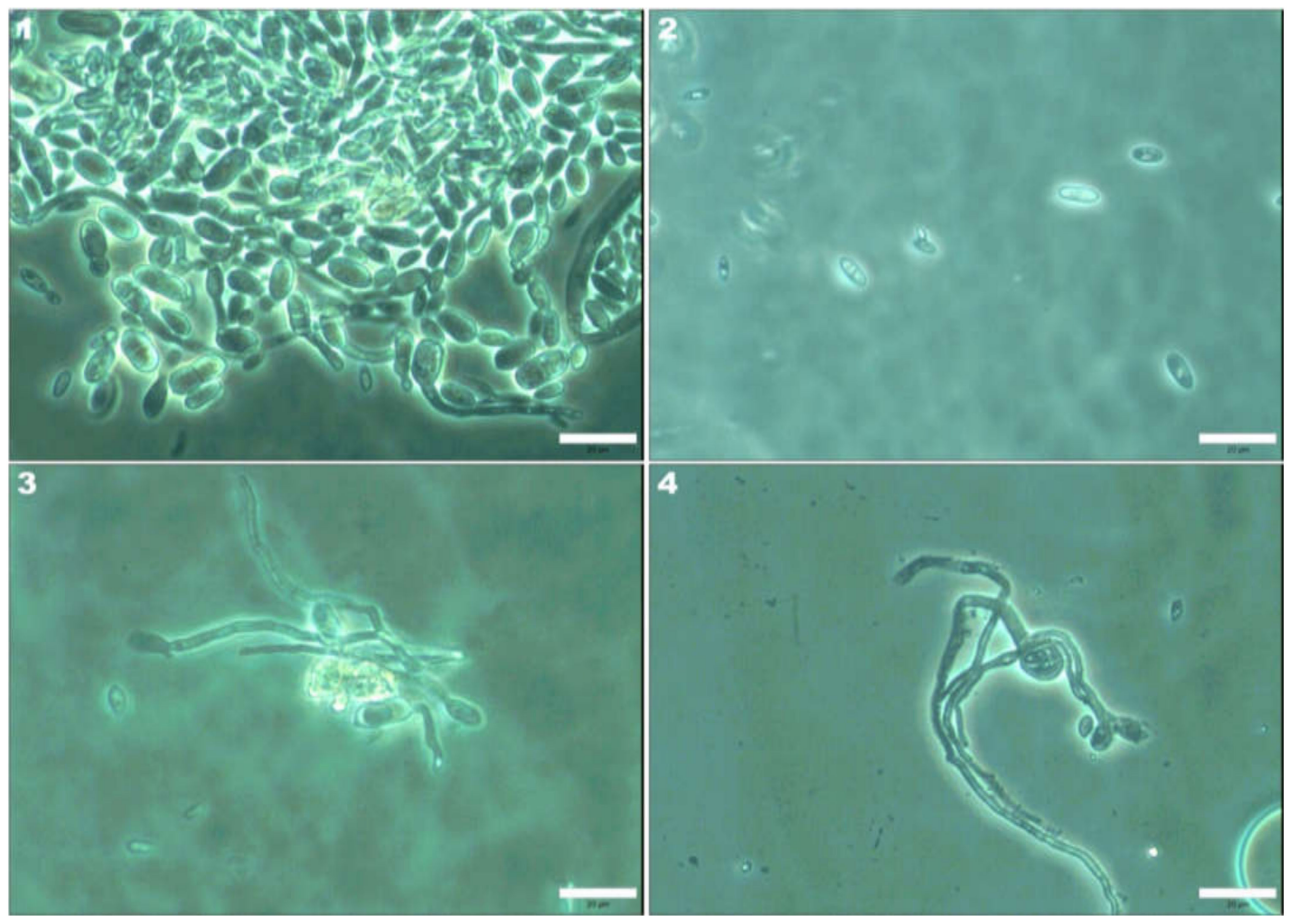
Morphology of *A. pullulans* cells observed by light microscopy: (**1**) control; (**2**) P-1; (**3**) P-2; (**4**) P-3 (scaling bar: 20 μm).

Special attention was paid to *A. pullulans* cells. This yeast-like fungus can be found in different environments: soil, water or air. *A. pullulans* cells show high adhesion abilities. This yeast-like fungus forms chlamydoconidia and blastoconidia with high environmental resistance, colonizes different surfaces and extensively produces extracellular polysaccharide pullulan (poly-α-1,6-maltotriose) [57]. Its biofilm and spore structures play an important role in protecting cells against adverse environmental conditions. For example, *A. pullulans* strains were able to inactivate some antimicrobials, namely antibiotics (virginiamycin) and disinfectants (phenols) [58,59]. However, in the case of *A. pullulans,* we observed altered morphology, especially after contact with both P-2 and P-3 surfaces (Figure 5). The cells showed abnormal elongated shapes in comparison to the control sample.

The results of cell-cycle disturbance of those dimorphic fungi in the presence of polymers are very interesting. Planktonic cell and mycelium morphological transitions are not clear. It was documented that *A. pullulans* morphology may be influenced by numerous environmental factors. The effects of some toxic substances, namely alcohols, on the morphology of *A. pullulans* were studied by Sevilla and co-workers [60]. These antimicrobials induced the transition from yeast-like cells to mycelium forms. A similar phenomenon was observed in our studies. Interestingly, only swollen cells and chlamydospores, not mycelial cells, are responsible for pullulan formation [61]. This fact is important because pullulan enhances cell adhesion abilities, and it may explain the poor cell aggregation and biofilm formation by hyphae forms on the P-2 and P-3 surfaces.

In vitro evaluating antimicrobial activity was based on the agar well diffusion method. Because of the fact that P-2 and P-3 are too viscous and, as such, could not be precisely dosed into the agar plates, we decided to test the activity of P-1 and a range of hydrophobic organosilicon compounds as well as substances used for modification of P-1 in order to obtain P-2 (cysteine) and P-3 (*n*-propylamine). Additional tests with Me_3_SiCH_2_CH_2_SCH_2_COOH and neat thioglycolic acid (a substrate in the synthesis of P-1 and Me_3_SiCH_2_CH_2_SCH_2_COOH) were also carried out.

The obtained results showed that almost all tested substances, except LPSQ, Me_3_SiVi, VMS-T11 (not grafted with bioactive groups), inhibited the growth of tested microorganisms (Table 2). Correlation of the activity of thioglycolic acid, Me_3_SiCH_2_CH_2_SCH_2_COOH and P-1 with respect to Me_3_SiVi and VMS-T11 indicate that the grafting of a bioactive molecule on organosilicon compounds indeed enhances the antimicrobial properties of the latter. On the other hand, the polymeric nature of materials such as P-1 makes more feasible practical use of antimicrobial species of low molecular weight.

**Figure 6:**
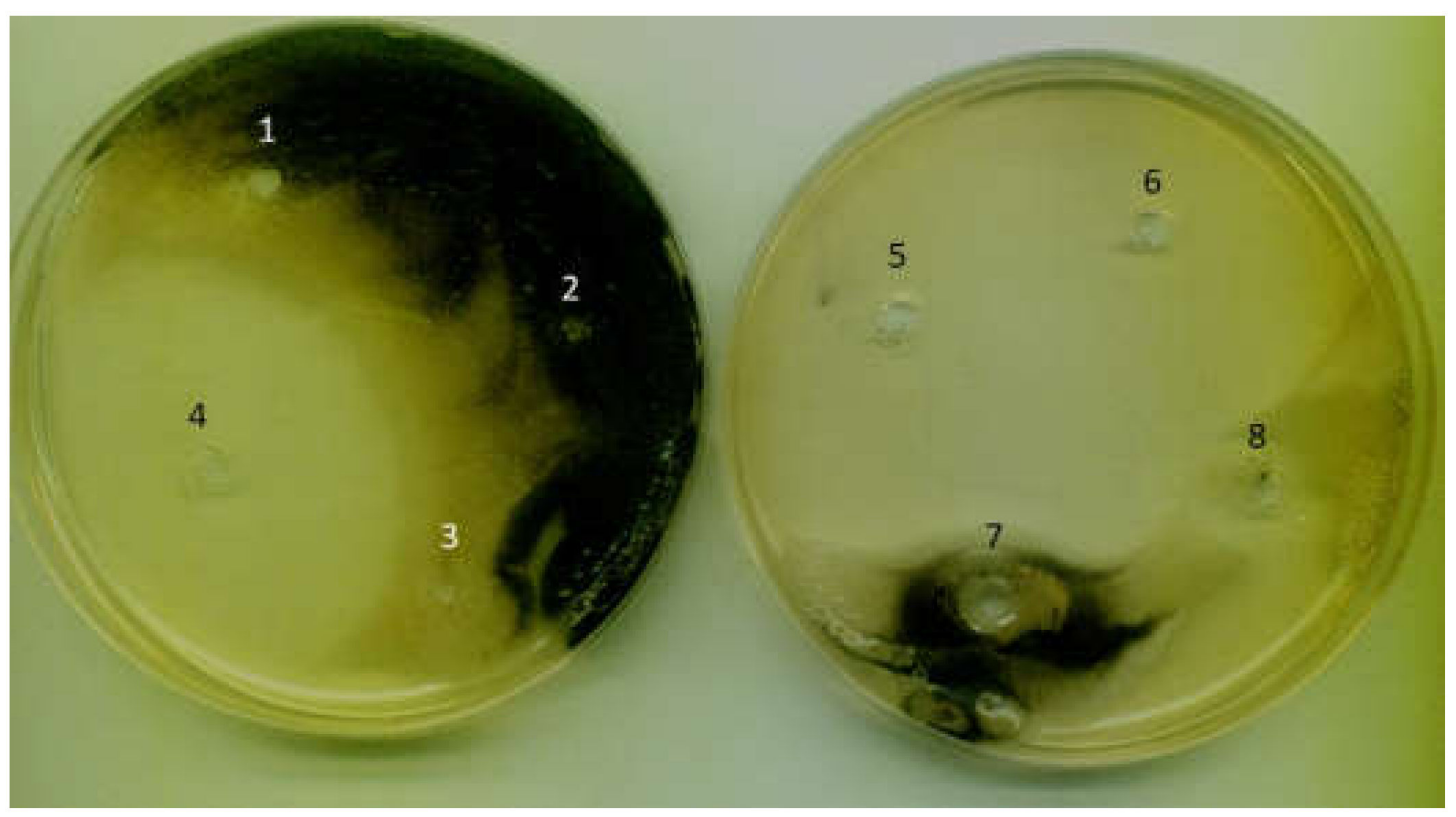
In vitro evaluation antimicrobial activity of selected chemicals on *A. pullulans* strain. (**1**) LPSQ; (**2**) Me_3_SiVi; (**3**) VMS-T11; (**4**) thioglycolic acid; (**5**) cysteamine; (**6**) *n*-propylamine; (**7**) Me_3_SiCH_2_CH_2_SCH_2_COOH; (**8**) P-1.

The strain *A. hydrophila* showed a high sensitivity to the compounds used. This feature could have influenced the best anti-adhesive effect of the functional polymers for this strain (Figure 4). In the case of the *A. pullulans* strain, the zones of growth inhibition were surrounded by a white mycelium area without dark pigmentation (Figure 6). This confirms that the tested antimicrobials markedly alter the growth cycle of these yeast-like fungi.

Therefore, our research into cell adhesion conducted during the 6-day incubation of yeast-like cells with bioactive compounds grafted as side chains on polysiloxane backbone as well as on the agar plates (agar well diffusion method) confirmed the antimicrobial and antiadhesive activities of tested functional polymers. Modified polymer surfaces are becoming more widely investigated for possible use in various settings including clinics, industry, and even the home. These results are promising but require further research on the mechanisms of possible cell destruction and biofouling inhibition by the new hydrophobic polysiloxanes. The future studies of the antimicrobial activity of polymers, either in solution or immobilized on surface, will provide interesting data and possibly new insight into the mechanisms of their antimicrobial action. It is worth paying attention to the long-term use of antimicrobial polymers and the potential risk of increasing microbial resistance.

## 3. Materials and Methods

### 3.1. Synthesis of Functionalized Polysiloxanes

The polymeric siloxane precursor—poly(vinylmethylsiloxane) terminated with trimethyl-silyl groups (VMS-T11; M_nGPC_ = 1800 g/mol, M_nNMR_ = 1000 g/mol, PDI = 1.3)—was purchased from Gelest (Morrisville, PA, USA). Other reagents: thioglyclic acid (≥99%); cysteamine (95%); *n*-propylamine ((≥99%); 2,2-dimethoxy-2-phenylacetophenone (DMPA, 99%) and silica (high-purity grade, pore size 60 Å, 70–230 mesh) were purchased from Sigma Aldrich (St. Louis, MO, USA) and used as received. Solvents (toluene (Chempur, Karlsruhe, Germany, pure p.a.) and tetrahydrofuran (THF, Chempur, pure p.a.)), were purified according to the literature procedures [62].

Trimethylvinylsilane (Me_3_SiVi, 97%), used for the in vitro tests of antimicrobial activity and for the synthesis of Me_3_SiCH_2_CH_2_SCH_2_COOH, was purchased from Sigma Aldrich and used as received. Poly(vinylsilsesquioxane) terminated with trimethylsilyl groups (LPSQ) was obtained as previously reported [50].

#### 3.1.1. Synthesis of poly[2-(carboxymethylthioethyl)methylsiloxane] (P-1)

The functionalized polysiloxane was prepared with 90% yield by thiol-ene addition of thioglycolic acid to vinyl groups of poly(vinylmethylsiloxane), adapting the earlier published procedure [32].

NMR (THF-d_8_) δ (ppm):

^1^H: 0.1 (-OSiMe_3_), 0.2 (-SiMe), 0.9 (-SiCH_2_-), 2.7 (-CH_2_S-), 3.2 (-SCH_2_-)

^13^C: −1.3 (-SiMe), 1.0 (-OSiMe_3_), 17.6 (-SiCH_2_-), 26.9 (-CH_2_S), 33.3 (-SCH_2_), 173.2 (-COOH)

^29^Si: −23.6, –21.3 (-CH_2_(Me)SiO_2/2_), 8.7 (-OSiMe_3_)

FTIR (cm^−1^): 3500–3000 (ν O-H), 3000–2560 (ν C-H), 1710 (ν C=O), 1418 (δ C-OH), 1381 (δ C-H), 1275 (ν C-O), 1261 (δ Si-C), 1172 (δ Si-CH_2_), 1087 (ν Si-O-Si), 798 (ρ Si-CH_3_)

Me_3_SiCH_2_CH_2_SCH_2_COOH was prepared following exactly the same procedure (except the ratio [-CH = CH_2_]_0_/[S-H]_0_ = 1) using trimethylvinylsilane as the substrate. The volatiles were removed under reduced pressure to leave liquid product (Y = 98%). The product was used without purification.

NMR (THF-d_8_) δ (ppm):

^1^H: 0.0 (-SiMe_3_), 0.85 (-SiCH_2_-), 2.7 (-CH_2_S-), 3.2 (-SCH_2_-)

#### 3.1.2. A General Procedure for the Synthesis of poly[2-(mercaptoethylamidomethylthioethyl)methylsiloxane] (P-2) and poly[2-(*n*-propylamidomethylthioethyl)methylsiloxane] (P-3)

P-2 and P-3 were obtained using P-1 as the substrate. A procedure of amidation carried out with the use of a heterogeneous catalyst (SiO_2_) was applied [33,34]. P-1 was charged under argon into a Schlenk flask and dissolved in freshly distilled THF (Table 3). Cysteamine dissolved in DMF (P-2) or *n*-propylamine (P-3, no additional solvent) was added to this solution, followed by the addition of toluene, and the mixture was stirred for 15 min at room temperature. SiO_2_ was added and the mixture was heated at 100 °C for one week. The reaction progress was monitored through FTIR analysis. When no changes in the intensity of the bands characteristic of C(O)OH (decrease in ν(C=O) IR band at around 1700 cm^−1^) and C(O)NH (increase in amide I band at 1650 cm^−1^ (ν C=O) and amide II band at 1578 cm^−1^ (δ NH in plane + ν C-N)) were observed, the reaction mixture was heated for another 24 h. FTIR spectrum showed no further changes and the reaction was terminated. Once the maximum possible amidation of carboxylic groups of P-1 was reached, the reaction mixture was cooled down and filtered. The filtrate was concentrated under reduced pressure and the product still containing a small amount of THF was purified by precipitation into pentane (THF/pentane). After separation, the product was reprecipitated from THF/diethyl ether and then MeOH/pentane. All products were very viscous liquids (semi-waxes). The described conversion of the carboxyl groups (approx. 70%) was the maximum value achieved under the described reaction conditions. The observed presence of free carboxyl groups resulted from their incomplete substitution.

##### P-2

NMR (THF-d_8_) δ (ppm):

^1^H: 0.2 (s. -SiMe + -OSiMe_3_), 0.9 (m. -SiCH_2_-), 2.7 (m. -CH_2_S-), 3.3 (m. -SCH_2_-), 3.5 (NH-CH_2_- + -CH_2_-SH), 3.43 (-SH), 8.2 (-NH-)

^13^C: −0.9 (-SiMe + -OSiMe_3_), 17.3 (-SiCH_2_-), 27.2 (-CH_2_SH), 33.2–35.0 (-SCH_2_-), 37.0–39.2 (-CH_2_S-), 40.6 (-NHCH_2_-), 170.2 (-C(O)NH-), 173.2 (COOH) (residue)

^29^Si: –23.4, –21.3 (-CH_2_(Me)SiO_2/2_), 10.0 (-OSiMe_3_)

FTIR (cm^−1^): 3500–3000 (ν O-H), 3287 (ν N-H), 3000–2940 (ν C-H), 2460 (ν S-H), 1700 (ν C=O) (carboxylic groups), 1660 amide I band (ν C=O), 1536 amide II band (δ NH in plane + ν C-N), 1381 (δ C-H), 1261 (δ Si-C), 1169 (δ Si-CH_2_), 1076 (ν Si-O-Si), 798 (ρ Si-CH_3_)

##### P-3

NMR (THF-d_8_) δ (ppm):

^1^H: 0.2 (-SiMe + -OSiMe_3_), 0.9–1.0 (-SiCH_2_- + -CH_3_), 1.7 (-CH_2_-CH_3_), 2.7 (-CH_2_S-), 3.2 (-SCH_2_- + -NHCH_2_-), 8.3 (-NH-) 

^13^C: 0.8 (-SiMe + -OSiMe_3_), 10.6 (-CH_2_-CH_3_), 17.6 (-SiCH_2_-), 20.8 (-CH_2_-CH_3_), 26.5 (-SCH_2_-), 35.4 (-CH_2_S-), 41.1 (-NHCH2-), 170.8 (-C(O)NH-), 174.6 (-COOH) (residue)

^29^Si: −23.5, −21.2 (-CH_2_(Me)SiO_2/2_), 8.6 (-OSiMe_3_)

FTIR (cm^−1^): 3500–3000 (ν O-H), 3250 (ν N-H), 3000–2500 (ν C-H), 1700 (ν C=O) (carboxylic groups), 1650 amide I band (ν C=O), 1578 amide II band (δ NH in plane + ν C-N), 1379 (δ C-H), 1264 (δ Si-C), 1164 (δ Si-CH_2_), 1080 (ν Si-O-Si), 798 (ρ Si-CH_3_)

### 3.2. Analytic Methods

#### 3.2.1. Nuclear Magnetic Resonance Spectroscopy (NMR)

Liquid state ^1^H-, ^13^C- and ^29^Si-NMR spectra for the functionalized polysiloxanes were recorded in THF-d_8_ as a solvent on a Bruker DRX-500 MHz spectrometer, with TMS as the reference (Billerica, MA, USA).

#### 3.2.2. Fourier Transform Infra-Red Spectroscopy (FT-IR)

The absorption spectra were recorded for thin films of samples cast on crystal KBr windows using a Nicolet 380 FTIR spectrometer (Thermo Scientific, Waltham, MA, USA). The spectra were obtained by adding 32 scans at a resolution of 1 cm^−1^. The position of characteristic vibration modes was correlated with the appropriate literature data [34,63].

#### 3.2.3. Thermogravimetric Analysis (TGA)

The thermal stability of the studied polymeric materials was analyzed with a Hi-Res TGA 2950 Thermogravimetric Analyzer (TA Instruments, New Castle, DE, USA) in nitrogen atmosphere (heating rate 10 °C/min, resolution 3, sensitivity 3).

#### 3.2.4. Differential Scanning Calorimetry (DSC)

The samples were studied using a DSC 2920 Modulated apparatus (TA Instruments, New Castle, DE, USA). Thermograms were taken for samples (sealed in aluminum pans) heated in N_2_ atmosphere at the rate of 10 °C/min from room temperature to 100 °C, ten cooled down at the same rate to −50 °C and heated again to 100 °C. The cooling/heating cycle was repeated and the temperatures of characteristic phase transitions were taken from the third measurement.

#### 3.2.5. Surface Energy Measurements

Thin polymer films were spread on commercially available clean glass slides (clean-ready to use, Lab Glass) and 3-aminopropyltriethoxysilane pre-treated glass supports (APTES-glass, silane-prep slides, Sigma Aldrich) using a slit coating applicator (film thickness of 150 μm). Surface free energy was estimated by contact-angle measurements (sessile drop technique) at the film–air interface, as described earlier [22], using deionized water and glycerol (Chempur, pure p.a., anhydrous) as the reference liquids. Surface energies (including their polar and dispersive components) were estimated by the Owens–Wendt method [64].

### 3.3. Biological Studies

#### 3.3.1. Biological Material

Reference strains *E. coli* ATCC 8738, and *S. aureus* ATCC 6538, as well as water-borne bacterial isolates *A. hydrophila* KC756842 and *A. tumefaciens* KJ719245 deposited at LOCK Culture Collection [65], were subcultured on Triptic Soy Agar slants (Merck Millipore, Burlington, MA, USA). In turn, mold *A. pullulans* LOCK0461 was cultivated on Malt Extract Agar slants (Merck Millipore). Tested microorganisms were cultivated at 28–30 °C for 48 h, and then maintained at 4 °C. For *C. vulgaris* incubation in liquid Bold Basal medium [66] at 25 °C for 7 days, the light was provided by a cool white LED (T5 15W 6400 K, 80 μmol·m^−2^·s^−1^) with continuous illumination within the experimental period.

#### 3.3.2. Abiotic Surfaces

Bacterial adhesion was evaluated under laboratory conditions. Samples of the studied polymers (P-1, P-2, P-3 and LPSQ) were cast on clean glass slides (clean, ready to use, Lab Glass) using a slit coating applicator (film thickness of 150 μm). Experiments were conducted using white glass as the control carrier (Star Frost 76 mm × 26 mm, Waldemar Knittel Glasbearbeitungs GmbH, Bielefeld, Germany). Glass carriers were sterilized by autoclaving at 121 °C, and modified carriers were sterilized using UV light (265 nm, 2 h per each side).

#### 3.3.3. Assessment of Bacterial Adhesion

Sterile carriers (10 mm × 10 mm) were placed in sterile 25 mL Erlenmeyer flasks with 20 mL of culture medium (Table 4).

The amount of cell inoculum (0.1 mL) was standardized densitometrically (1°McF). The samples were incubated at 25 °C with agitation on a laboratory shaker (135 rpm) for 6 days. For *C. vulgaris* incubation, the cool white LED (T5 15 W 6400 K, 80 μmol·m^−2^·s^−1^) with continuous illumination was used. Cell adhesion to the carriers was evaluated using both fluorescence microscopy and luminometry using ATP-free sampling pens (Merck KGaA, Darmstadt, Germany). Luminometric measurements were expressed in Relative Light Units (RLU) using a HY-LiTE2 luminometer (Merck KGaA, Darmstadt, Germany). The relative adhesion coefficient (A) was then calculated: the RLU result for adhered cells was divided by the RLU results for culture suspension in the given sample [67]. Adhered bacterial cells were observed after DAPI staining and using the fluorescence microscope NICON type BX41 fitted with a 50× lens and with top illumination of the tested surfaces by an external lamp. Images were captured with a digital camera.

#### 3.3.4. Determination of Antimicrobial Activity of Working Solutions Used to Create Functional Polymers and Other Model Compounds

The routine antimicrobial susceptibility testing was based on the agar well diffusion method [68]. The agar plate surface was inoculated by spreading 200 µL of the microbial inoculum over the entire agar surface (TSA (Merck KGaA, Darmstadt, Germany) for bacteria, MEB (Merck KGaA, Darmstadt, Germany) for fungi, bold basal agar for algae). Then, a hole with a diameter of 5 mm was punched aseptically with a sterile cork borer, and a volume of 10 µL of the tested substance at the desired concentration was introduced into the well. Then, agar plates were incubated at 25 °C. The antimicrobial agent diffused in the agar medium and inhibited the growth of the microbial strain tested. After incubation, the antimicrobial activity of the tested molecules was detected by the appearance of the inhibition zone (mm) around the well.

#### 3.3.5. Statistical Methods

The results of microbial adhesion were calculated as the means and standard deviations in the data from three independent tests. Analysis of variance (ANOVA) was used to examine the differences between group means, representing the adhesion results (OriginLab Corporation, Northampton, MA, USA). The results were compared to those for the control samples (glass carriers). Values with letters show statistically significant differences: a, *p* ≥ 0.05; b, 0.005 < *p* < 0.05; c, *p* < 0.005.

## 4. Conclusions

We have shown that specific functionalization of polysiloxanes may increase their antiadhesive properties against a range of prokaryotic and eukaryotic microorganisms of different cell organization and cell wall structures, while not decreasing the inherent hydrophobic nature of silicone materials. Polysiloxanes bearing 2-(carboxymethylthioethyl)-, 2-(*n*-propylamidomethylthioethyl)- and 2-(mercaptoethylamidomethylthioethyl)- side groups have shown good antimicrobial and antibiofilm activity towards selected strains of bacteria (*A. hydrophila*, *S. aureus*, *E. coli*), fungi (*A. pullulans*) and algae (*C. vulgaris*). The results obtained for model thin-coating samples are promising for application of the studied polymers incorporated as structural units in more complicated macromolecular systems, e.g., crosslinked silicone rubber or gels.

This research is in line with current trends and developments for antimicrobial materials. The antimicrobial and antiadhesive polymers not only play a critical role in cell biology, but they are a powerful tool in many applications, where the control of microbial adhesion is necessary. However, it is worth paying attention to the mechanisms of antimicrobial action of modified compounds, effects of their lasting action on microbial cells and whether the long-term use of such antimicrobial materials is not associated with the risk of increasing cell resistance to the biocides used.

## Figures and Tables

**Table 1:** Thermal degradation characteristics (the prepared materials and their precursor).

Sample	T_5%_ (°C)	T_d1_ (°C)	T_d2_ (°C)	T_d3_ (°C)	Rate (%∙min/°C)			Residue (%)
					V_d1_	V_d2_	V_d3_	
VMS-T11	87.6	117.2	272.0	554.1	8.41	4.59	0.38	10.1
P-1	137.6	135.6	350.3	506.5	0.30	5.77	0.34	33.4
P-2	135.4	134.1	238.2	357.4	0.81	6.10	2.77	32.7
P-3	129.7	159.5	256.7	361.9	1.73	0.71	5.80	33.3

T_d_—peak temperature of weight loss derivative; V—rate of weight loss at major decomposition step; T_5%_—5% weight loss temperature; Residue—char residue at 600 °C.

**Table 2:** Growth inhibition zones (mm) determined by agar well diffusion method.

Strain	1	2	3	4	5	6	7	8
*A. hydrophila*	0	0	0	35	38	40	18	16
*A. tumefaciens*	0	0	0	30	33	30	12	14
*E. coli*	0	0	0	10	23	29	11	10
*S. aureus*	0	0	0	28	27	27	8	10
*A. pullulans*	0	0	0	33	32	36	7	15
*C. vulgaris*	0	0	0	28	17	13	8	10

(1) LPSQ; (2) Me_3_SiVi; (3) VMS-T11; (4) thioglycolic acid; (5) cysteamine; (6) *n*-propylamine; (7) Me_3_SiCH_2_CH_2_SCH_2_COOH; (8) P-1.

**Table 3:** Composition of reaction mixtures during the synthesis of P-2 and P-3.

Sample	P-2	P-3
P-1 (g)	3	3
cysteamine (mol)	0.0168	-
*n*-propylamine (mol)	-	0.0168
[COOH]_0_/[NH_2_]_0_ THF (mL) toluene (mL) DMF (mL) SiO_2_ (g) X (%) Y (%)	1 10 90 45 0.4 72 56	1 10 80 - 0.4 63 52

Y—reaction yield; X—degree of conversion of -COOH groups into (-C(O)NH-).

**Table 4:** Culture media.

Kind of Microorganisms	Culture Medium	Reference
Bacteria	50-fold diluted buffered peptone water (Merck KGaA, Darmstadt, Germany)	[65]
Fungi		
Algae	Bold basal medium	[66]

## Data Availability

Data presented in this study are available on request.

## Sample Availability

Samples of the compounds are available from the authors only for scientific collaboration.
